# Monitoring the progress and impact of a multicountry, interdisciplinary research project on childhood stunting: the UKRI GCRF Action Against Stunting Hub MEL protocol

**DOI:** 10.1136/bmjpo-2023-002428

**Published:** 2024-07-20

**Authors:** Dinesh Yadav D M, Darius Tetsa Tata, Modou Lamin Jobarteh, Rose Ndulu Ndolo, Santosh Kumar Banjara, Little Flower Augustine, Munikumar Manne, Bharati Kulkarni, Assana Diop, Fassiatou Tairou, Camara Fatou Ndiaye Diop, Babacar Faye, Umi Fahmida, Min Kyaw Htet, Nur L Zahra, Arienta R P Sudibya, Francis Naab, Marie K Harder, Chelsey Victoria Knott, Hugh Sharma Waddington, Claire Heffernan

**Affiliations:** 1 Department of Population Health, London School of Hygiene & Tropical Medicine, London, UK; 2 Epidemiology and Population Health, London School of Hygiene & Tropical Medicine, London, UK; 3 Clinical Division, National Institute of Nutrition, Hyderabad, Telangana, India; 4 ICMR-National Institute of Nutrition, Hyderabad, India; 5 National Institute of Nutrition, Hyderabad, India; 6 Service de Parasitologie-Mycologie-Pédiatrie, Faculté de Médecine, Université Cheikh Anta Diop (UCAD), Dakar, Senegal; 7 Service de Parasitologie-Mycologie, Université Cheikh Anta Diop (UCAD), Dakar, Senegal; 8 Université Cheikh Anta Diop (UCAD), Dakar, Senegal; 9 Southeast Asian Ministry of Education Organisation Regional Centre for Food and Nutrition (SEAMEO RECFON), Central Jakarta, DKI Jakarta, Indonesia; 10 Research, SEAMEO Regional Center for Food and Nutrition, Jakarta Timur, DKI Jakarta, Indonesia; 11 Perivoli Africa Research Centre, University of Bristol, Bristol, UK; 12 Values and Sustainability Research Group, School of Architecture, Technology and Engineering, University of Brighton, Brighton, UK; 13 School of Environmental Science and Engineering, Fudan University, Shanghai, China; 14 Department of Disease Control, London School of Hygiene & Tropical Medicine, London, UK; 15 London School of Hygiene & Tropical Medicine (LSHTM), London, UK; 16 London International Development Centre (LIDC), London, UK

**Keywords:** Monitoring, Qualitative research, Data Collection

## Abstract

**ABSTRACT:**

**Introduction:**

Monitoring, Evaluation and Learning (MEL) is an integral part of research, programme and policy development and implementation. However, MEL methods used to monitor and evaluate interdisciplinary research projects are often informal and under-reported. This article describes the MEL protocol of the UKRI GCRF Action Against Stunting Hub (AASH).

**Methods and analysis:**

The AASH conducts interdisciplinary research into childhood stunting in India, Indonesia and Senegal across 23 distinct work packages. Project-specific MEL framework and methods will be implemented. A logframe will be developed to monitor and evaluate the research activities across the field sites including the number of participants recruited, questionnaires, measurements and procedures completed. MEL dashboards using Tableau and Glasscubes will be used to track and report progress, milestones and outcomes of the project. Dashboard outputs will be reported as numbers and percentages, with additional graphs/charts for easy visualisation. A ‘learning’ framework will be developed to outline appropriate pipelines for the dissemination of the research findings. This includes a theory of change explicating the overarching ambitions of the project in influencing policy, practice and research, and strategic engagement of relevant stakeholders to evaluate knowledge, attitudes and best practices for impactful engagement and dissemination of the research findings.

**Ethics and dissemination:**

Ethical approval was granted by the Ethics Committee of the London School of Hygiene & Tropical Medicine (17915/RR/17513); National Institute of Nutrition (ICMR)–Ministry of Health and Family Welfare, Government of India (CR/04/I/2021); Health Research Ethics Committee, University of Indonesia and Cipto Mangunkusumo Hospital (KET-887/UN2.F1/ETIK/PPM.00.02/2019); and the National Ethics Committee for Health Research (CNERS), Senegal (Protocole SEN19/78). Findings from this work will be published in peer-reviewed journals, presented in conferences and disseminated to policy makers and research communities.

**WHAT IS ALREADY KNOWN ON THIS TOPIC?**

**WHAT THIS STUDY HOPES TO ADD?**

- This protocol reports the MEL-specific components of a large, multicountry, interdisciplinary research project on childhood stunting.
- The paper details the MEL framework and methods for monitoring the progress of the research, evaluating equitable partnerships, the impact of capacity-strengthening activities and the levels and quality of stakeholder engagement.

**HOW THIS STUDY MIGHT AFFECT RESEARCH, PRACTICE OR POLICY**

- By detailing the MEL framework and methods of an interdisciplinary research project, this protocol hopes to add to the literature on interdisciplinary research management.

## Introduction

 The UKRI GCRF Action Against Stunting Hub (AASH)—a partnership of 18 institutions in India, Indonesia, Senegal and UK—was created to provide an interdisciplinary understanding of the many drivers of child stunting. The theoretical framework underpinning the research is referred to as the ‘Whole Child Approach’ (WCA). This approach focuses on the synergies and interactions of drivers across the ‘whole child’ which have the potential to modulate growth and development. The WCA assesses the role of the home environment, food systems, dietary intake, breastfeeding, childcare practices, early education, shared values and its mechanistic underlining including the role of gut health, inflammation, nutrition, epigenetics, microbiome, intestinal helminths and neurocognition. The research project comprised multisite, observational and intervention studies in India, Indonesia and Senegal, involving more than 2000 mother–infant pairs and collecting over 21 000 variables.

Inevitably, research of such magnitude requires a strong Monitoring, Evaluation and Learning (MEL) component. MEL is used across different projects to monitor specific activities, evaluate performance and impact of these activities and provide a space for learning by reflecting on the experience.^1^ In other words, MEL provides a framework for identifying problems/challenges, its underlining factors, and supports learning through sharing experiences, with a primary purpose of improving performance. In global health setting, MEL is used to keep oversight on research project or programme activities, ensure activities are conducted as per protocols or manuals, provide feedback on milestones and targets and identify and support underperforming areas. This is particularly important in large, longitudinal research projects involving mixed methods, multiple outcomes and interdisciplinary teams of researchers, conducted concurrently across different countries by teams with different capacities. MEL ensures the different sites and teams conduct the research to an acceptable standard, identify context-specific challenges and ensure teams are provided with additional support to fully implement the research activities.

Furthermore, a transformational global health research project should aim to have a legacy which transcends its immediate scientific purpose through transforming and improving research, policy, programme and practice in the research countries, and where possible, globally. Thus, the AASH aims to be transformational with a unique presence and legacy beyond its scientific objectives. The remit of MEL can, therefore, extend to include monitoring and evaluation of current programmes, policies and practices related to child stunting. This is important in identifying channels for influencing and augmenting the programmes, policies and practices. As such, MEL can interlink researchers, research communities and stakeholders ensuring that research findings lead to learning and praxis. MEL can also support wider project or programme goals regarding equitable partnerships and stakeholder engagement.

This paper describes the MEL protocol applied across the AASH. It reports on the methodology for development of a theory of change, monitoring and evaluating the progress of a multicountry, interdisciplinary research project, and stakeholder engagements, and outlines the contribution of the research project in influencing child nutrition policy and practice in the research countries and beyond.

## Methods and analysis

The AASH’s MEL framework aims to support and inform the planning, implementation and continuous evaluation of project progress across a wide partnership. The approach will be designed and refined through consultation with key project staff, and implemented through a network of in-country staff with oversight provided by the designated MEL officer.

The MEL framework focuses on five core areas of activities:

1. Research: monitoring and evaluation of research activities across the research countries.
2. Partnerships: evaluate and support equitable partnership throughout the project.
3. Capacity building: review and evaluation of capacity-strengthening activities and impacts.
4. Engagements/communication: review of public and stakeholder engagement activities.
5. Policy: understanding global and country-specific policies, programmes and practices around childhood stunting, and mapping pathways to effectively communicate the AASH research and findings to influence praxis.

Extensive consultation events will take place to inform in-country partners and researchers about the implementation of the MEL framework, data collection needs and the reporting system. The consultations will provide opportunities to resolve queries regarding the MEL Framework.

### The theory of change

**Figure 1:**
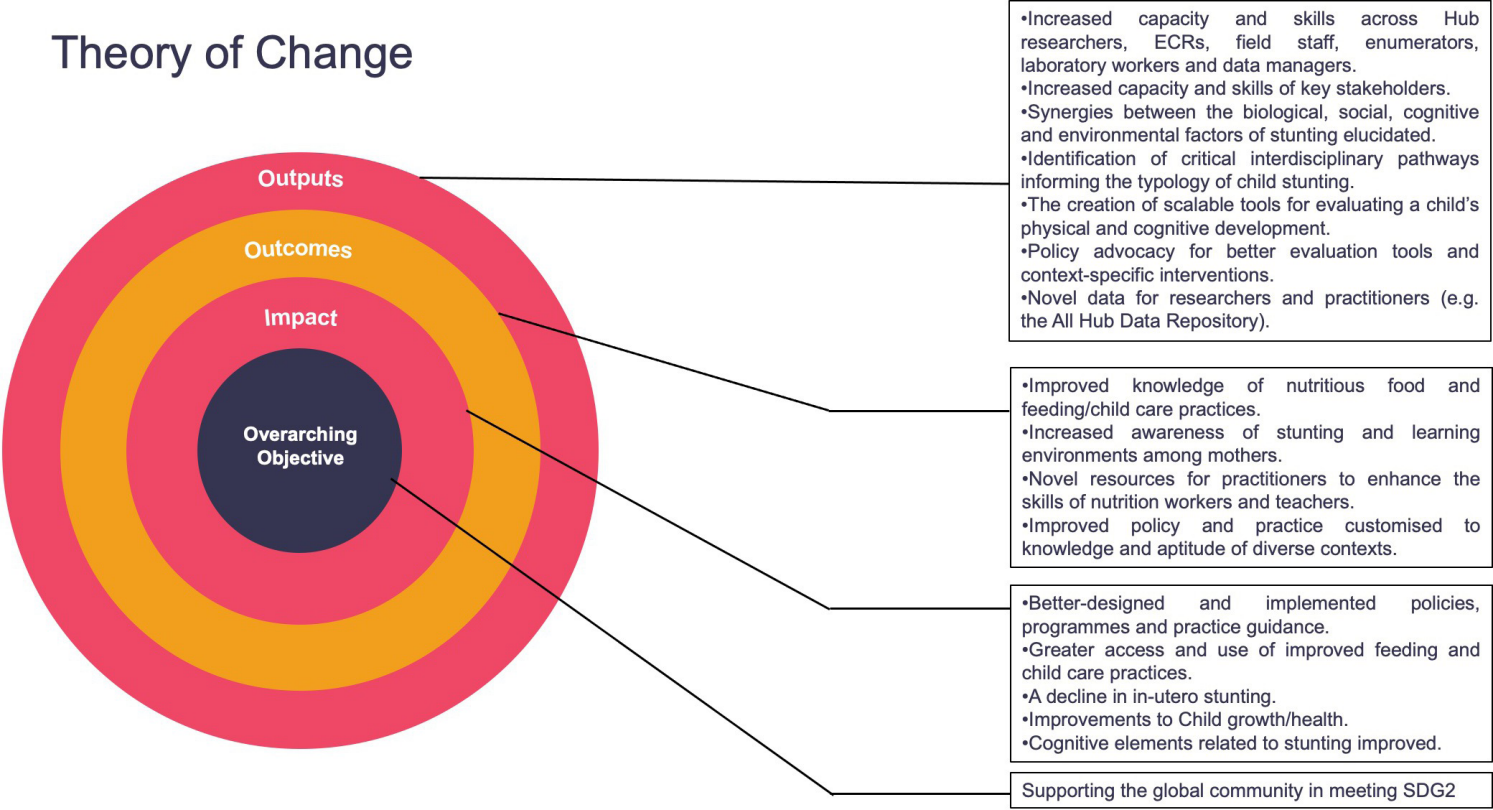
Theory of change of the Action Against Stunting Hub indicating its overarching objective, impact, outcomes and outputs. ECRs, early career researchers; SDG2, Sustainable Development Goal 2.

The development of a MEL strategy for AASH begins with a clearly defined theory of change.^2^ This involved mapping out the potential pathways to achieving the project’s overarching objective of supporting progress towards achieving Sustainable Development Goal 2, precisely its impacts on outcomes related to child stunting, influence on stakeholders and outputs of specific activities undertaken within the project. The theory of change was developed around the core thinking: *How can AASH contribute to the prevention and/or amelioration of childhood stunting and its associated outcomes, mitigate its long-term effects in communities in India, Indonesia and Senegal and contribute to better global policy and practice?* Through this thinking, palpable areas through which substantial changes can be achieved within the scope of the project were identified, including the opportunities around improving growth and development in utero, developing novel evaluation tools, supporting policy development and practitioner guidance, improving knowledge about and access to safe, nutritious foods, feeding practices and childcare, and capacity development across partners in the countries (figure 1). The evidence and assumptions underpinning this theory will be further examined within the research project.

### Operationalising the MEL framework

#### Developing the logical framework

Logical framework (also called logframe) will be used to map and track research activities. In developing the logframe, key research activities which required monitoring and evaluation will be identified including recruitment of study participants, sample and data collections in addition to the wider aspects of MEL, such as capacity development, equitable partnership, public and stakeholder engagements. Within the logframe, project performance indicators and associated timeframes (milestones) will be developed to monitor and evaluate progress, and provide accountability. The logframe will, thus, ensure accountability of the various actors and processes taking place.^3^ The logframe will be developed using Microsoft Excel to allow unrestricted access and use across the countries. The Excel spreadsheet allows logs of activities (activity log) with associated outcomes, outputs, performance indicators and means of verification (see online supplemental file 1). In this manner, the logframe will act as a systematic and quantifiable tool to track activities and the ability to meet the outputs and outcomes detailed in the theory of change. The completion of the logframe falls under the purview of the respective country managers in India, Indonesia and Senegal, and monitoring will be conducted progressively to ensure project activities are delivered.

#### Visualising progress

As noted above, AASH is being implemented by a wide range of partners and stakeholders across 23 work packages, hence, a mechanism to allow monitoring and evaluation of activities in ‘near real-time’ was deemed extremely valuable from the outset. Therefore, Tableau dashboards will be created to track and share progress both within and between country project management teams. The dashboards provide a mechanism of accountability and also facilitate the monitoring of activities across the different work packages of the AASH under a single platform.

**Figure 2:**
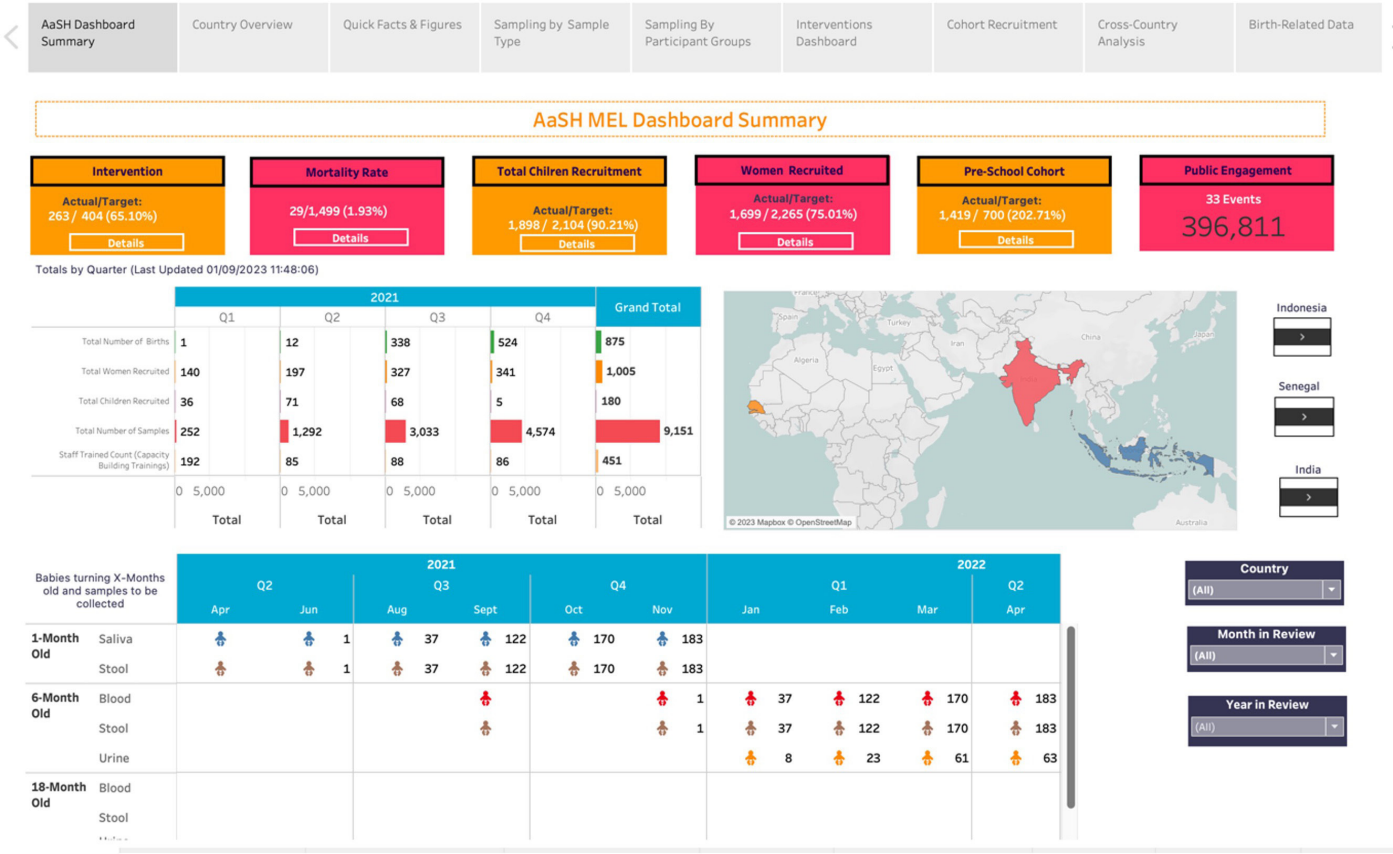
A sample image of the Monitoring, Evaluation and Learning (MEL) dashboard showing the progress of the different activities across the countries.

A user-centred design approach will be used to ensure the dashboards are demand driven and respond to the needs of the country management teams. Monitoring data will be entered into Glasscubes and linked to Tableau to build the interface. The dashboards have the advantage of interactive features including graphs and statistics to facilitate convenient visualisation of the research activities. The dashboards also have filters to allow users to visualise progress for specific activities, such as participants recruited, questionnaires completed and biological samples collected in numbers and percentages, allowing for effective planning and development of strategic contingencies where a field team is unable to meet project or workstream-specific milestones. This allows for effective planning and development of strategic contingencies where a field team is underperforming or unable to meet project or workstream-specific milestones. The dashboards will also provide a workspace to allow interactive discussion between research partners (figure 2).

#### Equitable partnerships

AASH aims to provide an accountable, fair and equitable working environment. Herein, equitable partnership involves the recognition and inclusion of the strengths and capacities individuals and organisations bring to an interdisciplinary project.^4^ But, to foster this shared responsibility, individuals and organisations need to recognise their own capacity and the added value they bring to the activities at hand. Working in an interdisciplinary research partnership across disciplines and at various scales often starts with co-development of the research question(s) by the various partners.^5^ AASH recognised this shared responsibility and involved partners in the development of the Hub’s theory of change, logical framework and work plans. Building and sustaining partnerships, however, takes time and can be broadened to include communities, local and national governments and other end users of the research. Equitable partnerships within the wider group provides a framework for shared responsibility based on mutual participation, trust and ownership. Thus, recognising and fostering equitable partnership is critical to sustaining cross-disciplinary endeavours such as policy and programme formulation.

Equitable partnership will be evaluated across the AASH through merging exercises and workshops to establish shared values and shared value action points.^6^ The workshops will be conducted to assess the evolution of shared values over time. The workshops will be conducted with the following objectives:

- Collectively define equitable partnerships.
- Identify shared metrics/indicators on which to monitor and evaluate equitable partnerships.
- Assess the performance of the Hub with regard to the shared value statements established at the beginning of the project.
- Realign the shared values of the Hub as the work progresses.

The data gathered from the workshops will be synthesised and a report produced with recommendations on how to foster equitable partnerships across AASH.

#### Capacity development

Capacity strengthening of partners is critical to the performance and success of all research projects. Thus, as a result, AASH will develop a detailed organogram (see online supplemental file 2) showing the various cadres of staff working on the project across the countries, strategic responsibilities of the staff and the balance of power, with a view to identify and strengthen capacity gaps. In addition, country-level roles across AASH will be organised based on a lateral-flow system to facilitate matching roles, where a role in one country has a similar role in the other countries, to promote a collaborative, peer-working and networking environment. Institutional twinning promotes power symmetry and plays a unique role in capacity development.^7^ The capacity development will be led by workstream leads and other notable experts who have a global presence and many years of experience in training people. Capacity strengthening in the initial phases of the Hub will be targeted to enhancing the skills of field staff, laboratory staff, early career researchers and the project management skills of the country teams. As the project progresses, capacity strengthening will focus on critical skills related to the particular themes from data and laboratory analysis. Workshops, seminars and round-table discussions will be used as the medium of training. In-country experts will be sought and added to the pool of trainers to ensure the trainings are tailored to the exact needs of attendees. Methods including survey and interviews will be used to evaluate the types of trainings offered, their quality and contribution to knowledge, attitudes and practices of attendees.

#### Stakeholder (public, private and policy) engagement

An important objective of the AASH is to inform policy and practice around childhood stunting. This will be achieved through series of targeted engagement with key stakeholders including academics, practitioners and programme and policy makers in India, Indonesia, Senegal, UK and globally. Policy influence is, however, a complex process determined by multiple factors beyond researchers and the delivery of research evidence.^8^ While the expertise of the researchers, research objectives and evidence are important in influencing policies, political context and characteristics of the policy issue have been found to determine which findings get taken up. These can operate through the three streams of public policy formulation—problem, policy and politics.

**Figure 3:**
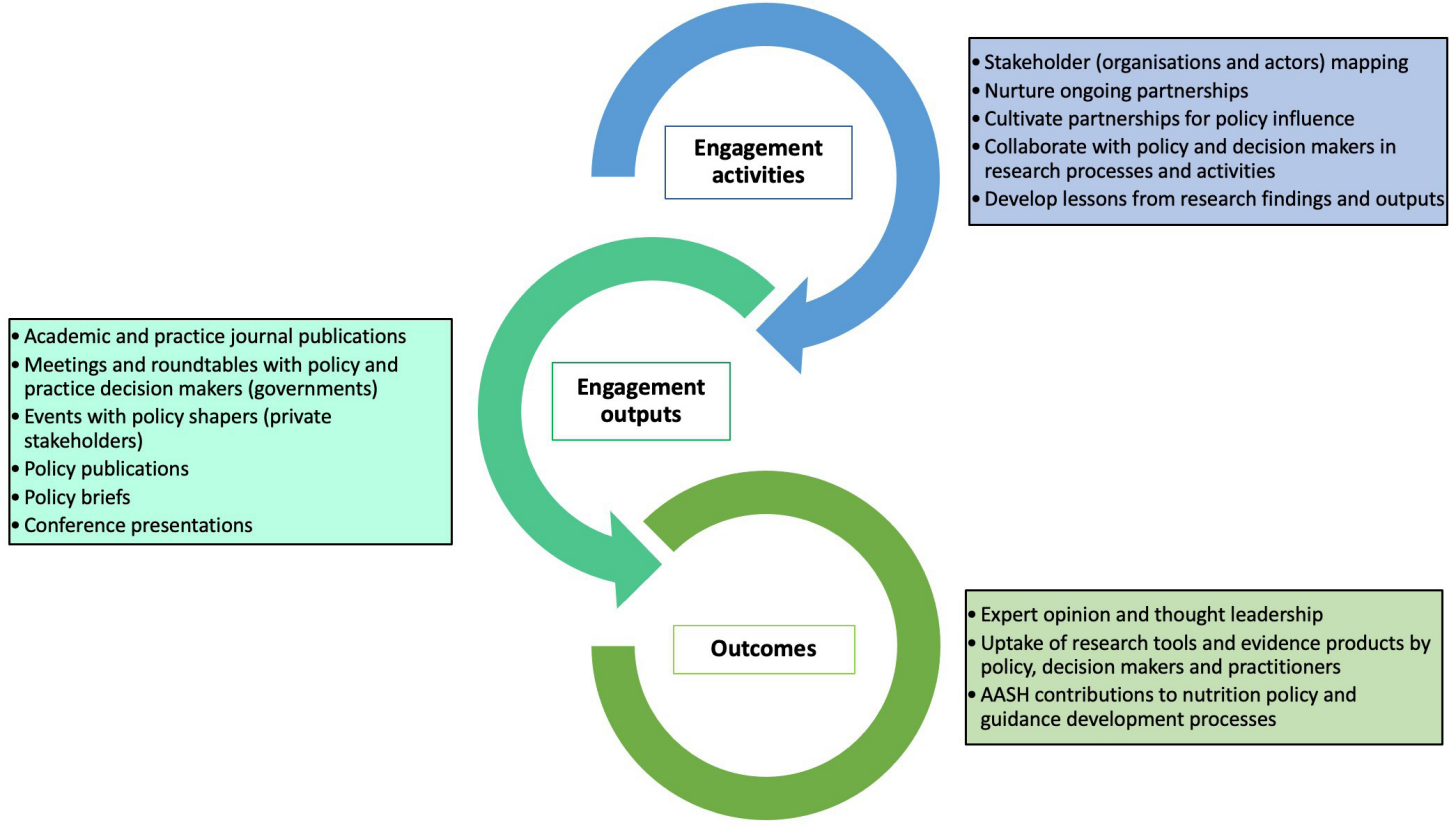
The policy engagement framework showing engagement activities, outputs and outcomes. AASH, Action Against Stunting Hub.

Research and policy processes often happen independently of each other with separate timelines, but intentional crossovers can be created through careful stakeholder engagement strategies.^9^ The implication of this phenomena is to intentionally explore and establish the crossovers between the research and policy processes.^10^ The primary objective is to develop appropriate, adaptable approaches to engage with relevant national and subnational stakeholders with the aim of augmenting or influencing policies, programmes and practice (figure 3).

With the knowledge that the timescale for generating interest and influence is key, AASH will use various mediums (including newspaper articles, radio broadcasts, billboards and outreach programmes) to stimulate interest in the research project in tandem with identifying, engaging and nurturing partnership with relevant stakeholders. A comprehensive engagement plan across different stakeholders in the countries will be developed, with an outlined objective for each engagement, mode of communication and outcomes (see online supplemental file 3). In general, a different cadre of stakeholders will be targeted, which can be classified into: (a) engaged participants, where collaboration and co-production will be the mode of engagement; (b) symbolic participants, where the mode of engagement is coordination and consultation; and (c) non-participants who will be engaged by outreach and communication (dissemination only).

Stakeholder map will be developed for each country with identification of the intersections of AASH research objectives and outcomes and country-specific policies and practices, and exploration of factors critical to successful implementation of policies and practices. A tracker will be developed to shape and track stakeholder engagement activities across the countries and institutions. Furthermore, teams in different countries and institutions will be encouraged to develop and implement their own engagement activities. The engagement strategy will be developed through consultation with the country partners and used to guide all engagement events across the AASH. All stakeholder engagements, including the number of people in attendance or reached, theme of the engagement and method of engagement, will be recorded in the engagement tracker and the output used in reporting the AASH overall engagements and reach.

### Data collection and analysis

The MEL framework includes a data collection and analysis plan component. This is important in facilitating accurate analysis and reporting of the progress in implementing the research project at the different sites, and assessment of the impact and reach of the AASH in terms of its ambition in equitable partnership, capacity strengthening, stakeholder engagements and dissemination of research findings, and other information related to childhood stunting through blogs, social media, newspaper articles and peer-reviewed journals. Logframe and dashboards will be used to capture quantitative data on the project activities and milestones, and report using statistical analyses of mean, percentage, SD and/or SEM. A mixed methods approach using both quantitative and qualitative data collection will be used in equitable partnership, capacity development and stakeholder engagement workshops. SPSS v.23 will be used to analyse the quantitative data derived from questionnaires and surveys. WeValue InSitu tool will be used to collect qualitative data during the workshop and analysed as described by Odii *et al*,^6^ and the NVivo qualitative data analysis software will be used for coding, categorising and identifying recurring themes in interview transcripts and open-ended survey responses.

## Ethics and dissemination

Ethical approval was granted by the Ethics Committee of the London School of Hygiene & Tropical Medicine (17915/RR/17513); the National Institute of Nutrition (ICMR)–Ministry of Health and Family Welfare, Government of India (CR/04/I/2021); Health Research Ethics Committee, University of Indonesia and Cipto Mangunkusumo Hospital (KET-887/UN2.F1/ETIK/PPM.00.02/2019); and the Comité National d’Ethique pour la Recherche en Santé, Senegal (Protocole SEN19/78). Results will be published in peer-reviewed journals and disseminated to policy makers and participating communities.

## supplementary material

10.1136/bmjpo-2023-002428 online supplemental file 1

10.1136/bmjpo-2023-002428 online supplemental file 2

10.1136/bmjpo-2023-002428 online supplemental file 3

## Data Availability

Data are available upon reasonable request.
