# Supplementary figures and images for: Monitoring the progress and impact of a multicountry, interdisciplinary research project on childhood stunting: the UKRI GCRF Action Against Stunting Hub MEL protocol
Source: BMJ Paediatr Open. 2024 Jul 20;8(Suppl 1):e002428. doi: 10.1136/bmjpo-2023-002428 (PMC11664339; doi:10.1136/bmjpo-2023-002428)

### Supplementary 3: AASH Organigram

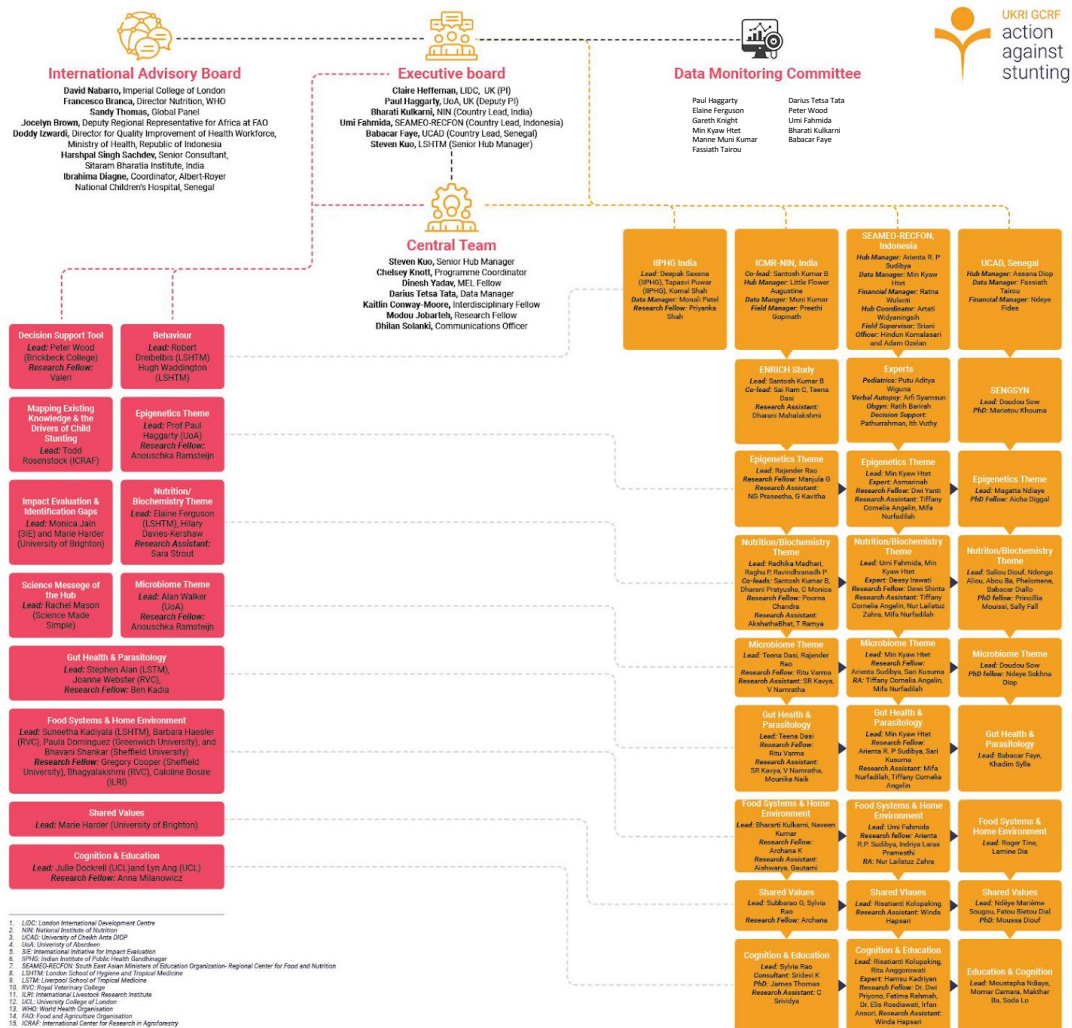

Supplement: online supplemental file 2 [file bmjpo-8-Suppl_1-s002.pdf]
