# Supplementary material for: Monitoring the progress and impact of a multicountry, interdisciplinary research project on childhood stunting: the UKRI GCRF Action Against Stunting Hub MEL protocol
Source: BMJ Paediatr Open. 2024 Jul 20;8(Suppl 1):e002428. doi: 10.1136/bmjpo-2023-002428 (PMC11664339; doi:10.1136/bmjpo-2023-002428)
Supplement: online supplemental file 3 [file bmjpo-8-Suppl_1-s003.docx]

Supplementary 2: Indicative stakeholder mapping

| **Stakeholder Group (Audience)** | **Objective/Purpose** | **Strategy/Media** |
| --- | --- | --- |
| National Planning Commissions  Poshan Abhiyan -India  Director of Nutrition -Indonesia  Cellule de Lutte contre la Malnutrition -Senegal | Information from Study observations  Policy recommendations | Reports, meetings and communications, academic outputs (papers, presentations), webinars/seminars |
| Ministry of Health, Maternal and Child Health | Administrative/ Operational support  Study observations  Policy recommendations | Meetings  Yearly reports  Policy recommendations |
| Regional and District Health Administration | Administrative/ Operational support  Study Observations | Meetings  Involvement in study operations  Reports |
| Ministry of Education | Administrative/ Operational support  Study Observations  Policy recommendations | Meetings with District level officers  Reports |
| Funders | Detailed information and comparable outputs; opportunities to provide feedback | Detailed written evaluation report (in addition to monitoring, research and finance reports as outlined above); follow-up for feedback |
| Academic/Research Organisations | Wider Collaborations | Meetings  Conferences  Webinars  Workshops  Publications |
| State-owned and Private media | Communicate outcomes of the study, specific topics relevant to public interest. Influence policy makers. | Newspaper articles  TV/radio interviews  Media briefings, Press conferences and  interviews with researchers, invitations to events |
| NGO’s, UN and other Multi-& Bi-lateral Aid Organisations | Strategic partnerships/uptake of Hub outputs | Meetings, conferences, joint stakeholder engagement activities. |
| Communities in Study Locations | Sensitisation re: Hub work and the information of the Study | Participant information sheets, Videos, Artwork and other multimedia. Community Caravans  Community Radios |
| Wider audiences and public | Sensitisation re: malnutrition and child stunting | Community Caravans  Community Radios  Local TV  Twitter, Facebook and LinkedIn |
