# Supplementary material for: Monitoring the progress and impact of a multicountry, interdisciplinary research project on childhood stunting: the UKRI GCRF Action Against Stunting Hub MEL protocol
Source: BMJ Paediatr Open. 2024 Jul 20;8(Suppl 1):e002428. doi: 10.1136/bmjpo-2023-002428 (PMC11664339; doi:10.1136/bmjpo-2023-002428)
Supplement: Supplementary file 4 [file bmjpo-2023-002428.reviewer_comments.pdf]

PEER REVIEW HISTORY

BMJ Paediatrics Open publishes all reviews undertaken for accepted manuscripts. Reviewers are asked to complete a checklist review form and are provided with free text boxes to elaborate on their assessment. These free text comments are reproduced below.

ARTICLE DETAILS

**TITLE (PROVISIONAL)**	Monitoring the progress and impact of a multi-country, interdisciplinary research project on childhood stunting: the UKRI GCRF Action Against Stunting Hub MEL protocol
**AUTHORS**	Yadav, Dinesh<br>Tata, Darius<br>Jobarteh, Modou<br>Ndulu Ndolo, Rose<br>Banjara, Santosh Kumar<br>Augustine, Little Flower<br>Munikumar, Manne<br>Kulkarni, Bharati<br>Diop, Assana<br>Tairou, Fassiatou<br>Fatou Ndiaye Diop, Camara<br>Faye, Babacar<br>Fahmida, Umi<br>Htet, Min Kyaw<br>Zahra, Nur<br>Sudibya, Arienta R.P.<br>Naab, Francis<br>Harder, Marie<br>Knott, Chelsey<br>Waddington, Hugh Sharma<br>Heffernan, Claire

VERSION 1 – REVIEW

**REVIEWER**	Dr. Pedro Almirall<br>Centro Municipal de Higiene y Epidemiología<br>Epidemiología<br>Calle C entre 29 y Zapata<br>La Habana<br>10400<br>United Kingdom of Great Britain and Northern Ireland
**REVIEW RETURNED**	04-Jun-2024

**GENERAL COMMENTS**	Thank you for the opportunity.<br>Monitoring the progress and impact of a multi-country interdisciplinary<br>research<br>project on childhood stunting: the UKRI GCRF action against stunting Hub MEL<br>protocol.<br>Authors: Dinesh Yadav DM1, Darius Testa Tata1 et al.<br>When reviewing this document, the first thing that should be noted is the<br>existence of few<br>studies on monitoring, evaluation and learning that include several countries, institutions,

	various disciplines and researchs.<br>India, Indonesia, Senegal and the UK are participating; 18 institutions, 23<br>work packages;<br>21000 variables will be obtained and 2000 mothers/children will be surveyed<br>which makes<br>equity between the participants the most important link when collecting<br>data and<br>analyzing them as well as between the participating research teams as it is<br>well clarified<br>in the different sections of the project.<br>This proposal is based on the “Four approaches to supporting equitable<br>research<br>partnerships in UKCDR and ESSENCE. Good practice document (2022) UK<br>collaborative<br>on development research" (Gaery Aslanyan, A.C.E-A et al.) which is one of<br>the guiding<br>documents for the development of multi-country equitable research studies<br>and which<br>reflects the experience of these two organizations in the equitable principles between<br>partners for this type of design.<br>The project is well structured since they took into account the difference<br>between<br>information systems between countries, the way of obtaining the data, the<br>selection of<br>appropriate indicators common to the participants in the research, a good<br>selection of<br>where to direct the funds, clarifying roles and responsibilities in each case were taken into<br>account. They also developed the aspects of the five core areas of activity<br>of the MEL<br>network: research, partnerships, building capacity, engagement/communication and<br>policies.<br>MEL and its specific components are not very commonly reported limiting<br>their ability to<br>use, hence the accurate of what this project proposes to provide with its<br>results:<br>1. Large-scale, multi-country MEL data and research projects on malnourished children.<br>2. Detailed reports of the created MEL network and methods for evaluating<br>the progress,<br>impact and strengthening of activities and their quality.<br>It would be recommended to include the analysis method to be used to evaluate the

	qualitative surveys

VERSION 1 – AUTHOR RESPONSE

Reviewer: 1
Dr. Pedro Almirall, Centro Municipal de Higiene y Epidemiología
Comments to the Author

Thank you for the opportunity.
Monitoring the progress and impact of a multi-country interdisciplinary research project on childhood stunting: the UKRI GCRF action against stunting Hub MEL protocol.

When reviewing this document, the first thing that should be noted is the existence of few studies on monitoring, evaluation and learning that include several countries, institutions, various disciplines and researchers-India, Indonesia, Senegal and the UK are participating; 18 institutions, 23 work packages; 21000 variables will be obtained and 2000 mothers/children will be surveyed which makes equity between the participants the most important link when collecting data and analyzing them as well as between the participating research teams as it is well clarified in the different sections of the project.
This proposal is based on the "Four approaches to supporting equitable research partnerships in UKCDR and ESSENCE. Good practice document (2022) UK collaborative on development research" (Gaery Aslanyan, A.C.E-A et al.) which is one of the guiding documents for the development of multi-country equitable research studies and which reflects the experience of these two organizations in the equitable principles between partners for this type of design.
The project is well structured since they took into account the difference between information systems between countries, the way of obtaining the data, the selection of appropriate indicators common to the participants in the research, a good selection of where to direct the funds, clarifying roles and responsibilities in each case were taken into account. They also developed the aspects of the five core areas of activity of the MEL network: research, partnerships, building capacity, engagement/communication and policies. MEL and its specific components are not very commonly reported limiting their ability to use, hence the accurate of what this project proposes to provide with its results:
1. Large-scale, multi-country MEL data and research projects on malnourished children.
2. Detailed reports of the created MEL network and methods for evaluating the progress, impact and strengthening of activities and their quality.
It would be recommended to include the analysis method to be used to evaluate the qualitative surveys

RESPONSE
We have now reviewed the manuscript and provided more details on the statistical methods. See lines 264-272.
Summary is given below
• We utilize bespoke statistical software (SPSS) to analyze quantitative metrics, including questionnaires and surveys to capture data related to participant ratings or understanding of various outcomes of interest.
• For qualitative data, we use the WeValue Insitu® tool during workshops, analyzed as described by Benita et al. 2024 (cited in the manuscript).
• Additionally, for qualitative analysis, we will employ NVivo software for coding, categorizing, and identifying recurring themes in interview transcripts and open-ended survey responses.
